# Regional Biases in MODIS Marine Liquid Water Cloud Drop Effective Radius Deduced Through Fusion With MISR

**DOI:** 10.1029/2019JD031063

**Published:** 2019-12-08

**Authors:** Dongwei Fu, Larry Di Girolamo, Lusheng Liang, Guangyu Zhao

**Affiliations:** ^1^ Department of Atmospheric Sciences University of Illinois at Urbana‐Champaign Urbana IL USA; ^2^ Science Systems and Applications, Inc. Hampton VA USA

**Keywords:** MISR, MODIS, effective radius, liquid clouds, bias‐correction, data fusion

## Abstract

Satellite measurements from Terra's Moderate Resolution Imaging Spectroradiometer (MODIS) represent our longest, single‐platform, global record of the effective radius (*Re*) of the cloud drop size distribution. Quantifying its error characteristics has been challenging because systematic errors in retrieved *Re* covary with the structural characteristics of the cloud and the Sun‐view geometry. Recently, it has been shown that the bias in MODIS *Re* can be estimated by fusing MODIS data with data from Terra's Multi‐angle Imaging SpectroRadiometer (MISR). Here, we relate the bias to the observed underlying conditions to derive regional‐scale, bias‐corrected, monthly‐mean *Re*_*1.6*_, *Re*_*2.1*_, and *Re*_*3.7*_ values retrieved from the 1.6, 2.1, and 3.7 μm MODIS spectral channels. Our results reveal that monthly‐mean bias in *Re*_*2.1*_ exhibits large regional dependency, ranging from at least ~1 to 10 μm (15 to 60%) varying with scene heterogeneity, optical depth, and solar zenith angle. Regional bias‐corrected monthly‐mean *Re*_*2.1*_ ranges from 4 to 17 μm, compared to 10 to 25 μm for uncorrected *Re*_*2.1*_, with estimated uncertainties of 0.1 to 1.8 μm. The bias‐corrected monthly‐mean *Re*_*3.7*_ and *Re*_*2.1*_ show difference of approximately +0.6 μm in the coastal marine stratocumulus regions and down to approximately −2 μm in the cumuliform cloud regions, compared to uncorrected values of about −1 to −6 μm, respectively. Bias‐corrected *Re* values compare favorably to other independent data sources, including field observations, global model simulations, and satellite retrievals that do not use retrieval techniques similar to MODIS. This work changes the interpretation of global *Re* distributions from MODIS *Re* products and may further impact studies, which use the original MODIS *Re* products to study, for example, aerosol‐cloud interactions and cloud microphysical parameterization.

## Key Points

- Regional monthly mean MODIS *Re* is biased high by at least 1 to 10 μm and depends on cloud horizontal heterogeneity and Sun angle
- Bias‐corrected values of *Re* compare favorably to other independent satellite retrievals, field observations, and global model simulations
- Spectral channel differences in bias‐corrected *Re* is reduced but still suggests that vertical variability of *Re* is cloud‐type dependent

## 1 Introduction

The effective radius (*Re*) of the cloud drop size distribution plays an important role in the energy and water cycle of the Earth system (Platnick & Twomey, 1994; Twomey, 1991) and is listed as an Essential Climate Variable by the Global Climate Observing System (GCOS, 2011). Satellite‐retrieved *Re* has extensively been used, for example, to assess aerosol‐cloud interactions (e.g., Menon et al., 2008) and to evaluate cloud parameterization in climate models (e.g., Ban‐Weiss et al., 2014; Suzuki et al., 2013). Owing to its importance, stringent requirements on *Re* accuracy of better than 10% have been called for (e.g., Ohring et al., 2005). However, comparison between currently available satellite‐derived *Re* products (e.g., Bréon & Doutriaux‐Boucher, 2005; Sayer et al., 2011; Stubenrauch et al., 2012) reveal disparities ranging from ~2 μm (~20%) to ~9 μm (~50%) in regional monthly‐mean values.

The *Re* products from the Moderate Resolution Imaging Spectroradiometer (MODIS) are widely used owing to its global coverage and long record—now over 18 years. MODIS uses two shortwave spectral channels to retrieve *Re* and cloud optical depth simultaneously (Platnick et al., 2003). This bispectral retrieval technique makes several assumptions, including 1‐D radiative transfer, single‐mode drop size distribution, and cloud horizontal and vertical homogeneity. Deviations from these assumptions in nature lead to potential bias in the retrieved *Re*. For example, Marshak et al. (2006) showed that the deviations from the 1‐D radiative transfer assumption can lead to overestimates of retrieved *Re* of up to a factor of 2 for simulated cumulus clouds, while Werner et al. (2016) showed case studies in which the difference between the MODIS *Re* and that deduced using a coincident higher‐resolution imager can exceed 10 μm for partially cloudy pixels—but with significant scatter having both positive and negative biases. Comparison between MODIS‐retrieved *Re* and in situ observations shows that overestimates in MODIS *Re* range from about −0.22 to 13 μm, depending on cloud type, Sun‐view geometry, the choice of in situ probe, and the details of the cloud microphysical properties (e.g., Ahn et al., 2018; Glienke et al., 2017; King et al., 2013; Painemal & Zuidema, 2011; Witte et al., 2018). In particular, Witte et al. (2018) suggested that while satellite retrievals are commonly validated against in situ measurements, the uncertainty of the aircraft retrievals should be acknowledged. They showed that in situ measurements of the MODIS *Re* bias may be overestimated. Nevertheless, these and many other studies presented tremendous insight into the issues in retrieving *Re* in the face of cloud heterogeneity, as well as in the quality of MODIS‐retrieved *Re* under certain conditions, none characterized the MODIS *Re* bias at regional scales over the entire globe. Recently, progress was made in Liang et al. (2015), where they characterized the latitudinal bias in MODIS‐retrieved *Re* through data fusion of the Multi‐angle Imaging Spectroradiometer (MISR; Diner et al., 1998) and MODIS, both onboard the Terra satellite. They showed that the zonal‐mean bias (i.e., averaged across longitude at a given latitude) in MODIS‐retrieved *Re* for marine liquid clouds varied from 2 to 11 μm, with smaller biases occurring at latitudes where stratiform clouds contribute the most to total cloud fraction.

In this study, we extend the approach of Liang et al. (2015) to estimate the monthly‐mean bias in the MODIS‐retrieved *Re* at regional scales. We develop a bias‐correction method that relates the bias to observed cloud properties and Sun‐view geometry and apply it to 8 years (2001–2008) of January and July MODIS‐retrieved *Re* from the 1.6, 2.1, and 3.7 μm spectral channels, respectively, namely, *Re*_*1.6*_, *Re*_*2.1*_, and *Re*_*3.7*_. We show that the differences among these three bias‐corrected *Re* products are greatly reduced compared to the original products and that the remaining differences may indicate vertical variations of *Re* that appears to be cloud‐type dependent. Comparisons between bias‐corrected *Re* and those derived from other satellite instruments, field observations, and climate models are also discussed.

## 2 Data and Method

Fusion of the Terra MISR and MODIS data at cloud top is described in detail in Liang et al. (2009) and Liang and Di Girolamo (2013). We use the MODIS Collection 6 Cloud Product (MOD06; Platnick et al., 2015) and Version 24 of the MISR L1B2 ellipsoid‐projected radiance product from 2001 to 2008 for the months of January and July in order to provide ample sampling for good statistical characterization and to contrast two opposing seasons. Only liquid water clouds were considered based on the cloud phase flag in MOD06. Cloud optical depth (*τ*) at the MISR view geometries are retrieved using MISR near‐infrared BRF and MODIS‐retrieved *Re* as described in Liang et al. (2009). The fusion between MISR and MODIS data requires projecting MODIS *Re* and *τ* retrievals onto the 1.1 km MISR SOM (Space Oblique Mercator) grid. To reduce registration errors of clouds across MISR cameras, pixels are grouped in 3 by 3 1.1 km pixel domains, followed by the M2 feature matcher (Muller et al., 2002) to track clouds across MISR's nine cameras. The quality control procedure requires that a given 3 by 3 pixel domain has successful coregistration across MISR's nine camera views and that valid MODIS *Re* and τ retrievals are identified as fully cloudy for the 3 by 3 domain (see Liang et al., 2009 for details on the quality control procedures, with further discussion in Liang & Di Girolamo, 2013). Following the quality control procedures, a total of 48.2% and 51.4% of all fully cloudy, valid MODIS retrievals are used in this study for January and July, respectively. The distribution of these MODIS *Re* values compared to the total population of MODIS *Re* values was shifted about 1 μm larger, but with no discernible differences in the spatial pattern. The distribution of τ for these data shows no shift in the mode of the distribution, but rather a narrowing of the distribution relative to the total population of MODIS τ values, particularly for large τ values that are often found at high latitudes in the winter hemisphere.

Our estimate of the *Re* bias in the MODIS products begins with the approach described in Liang et al. (2015). In brief, Liang et al. (2015) showed that cloud optical depth retrievals as a function of observed scattering angle exhibit a local minimum in the vicinity of the cloud‐bow scattering angle (~140°) whenever the *Re* is overestimated. They revealed that these local minima, referred to as τ‐rainbow dips (although more appropriately τ‐cloudbow dips), are prevalent within the MODIS cloud optical depth product, as well as in the MISR cloud optical depth retrievals that use MODIS *Re* as an input. They demonstrated that the amplitude of the τ‐rainbow dip can be used to estimate the magnitude of the *Re* bias in the MODIS products. However, depending on which side of the cloud bow is used to measure the amplitude of the τ‐rainbow dip (e.g., 135–140° vs. 140–145°), the bias is either overestimated or underestimated because of broader variations in the view‐angle dependence of retrieved cloud optical depth (e.g., Liang & Di Girolamo, 2013). Owing to MISR's unique ability to sample scattering angles on either side of the rainbow at many latitudes, they were able to produce upper and lower bound estimates of the MODIS *Re* bias at those latitudes, showing latitudinal variations in zonal‐mean *Re* that were consistent with expected biases caused by the departure from the homogeneous cloud assumption for clouds typically found at the observed latitudes (e.g., latitudes dominated by stratiform vs. cumuliform clouds). A separate study examined the impact of the vertical variation in *Re* and drizzle on the method of Liang et al. (2015) for estimating the bias in MODIS‐retrieved *Re*, showing that these two factors contribute no more than ~1 μm to the bias estimates over a wide range of drizzle and vertical variations in *Re* (Su, 2017). The potential impact of a fixed cloud droplet size distribution on the *Re* retrievals is also another source of error. As shown in previous studies (e.g., Benas et al., 2019; Bréon & Doutriaux‐Boucher, 2005; Chang & Li, 2002), the error in retrieving *Re* from the bispectral technique, such as used by MODIS, due to deviations from the assumed effective variance of the droplet size distribution is typically less than ~1 μm. The differences in the scattering angular variation in retrieved *Re* across the cloud bow (important to the method of Liang et al., 2015) between a range of effective variance are even smaller. This error does propagate to our results and, as shown below, is small relative to the difference between upper and lower bound estimates of the MODIS *Re* bias.

Here, we relate the upper and lower bound estimates of the MODIS *Re* bias had by the method of Liang et al. (2015) to other variables that can be measured by MODIS alone. This provides the basis for constructing a MODIS *Re* bias‐correction procedure, which we then apply to MODIS data to obtain regional estimates of bias‐corrected *Re* of marine liquid water clouds. The choice of variables is based upon our current understanding of potential factors that may contribute to the *Re* bias due to three dimensional (3‐D) radiative effects: solar‐zenith angle (*SZA*), cloud heterogeneity, and cloud optical depth (e.g., Grosvenor & Wood, 2014; Loeb & Davies, 1996; Marshak et al., 2006; Zhang et al., 2012). We did examine additional variables, such as cloud top height, cloud top temperature, and above‐cloud water vapor, but they showed no significant relationship to the observed MODIS‐retrieved *Re* bias (not shown).

The fused MISR‐MODIS data are stratified into five MISR nadir τ bins (0–4, 4–12, 12–16, 16–24, and >24) and seven cloud heterogeneity‐metric (*H*_*σ*_) bins (0–0.02, 0.02–0.04, 0.04–0.06, 0.06–0.08, 0.08–0.1, 0.1–0.2, and >0.2). These bin widths were chosen to loosely provide similar number of samples in each bin and to ensure good separation between the optically thin and smooth clouds (characterized by small τ and *H*_*σ*_) from the optically thick and rough clouds (characterized by large τ and *H*_*σ*_). Starting with MODIS Collection 6 (Platnick et al., 2015), *H*_*σ*_ is defined as the standard deviation divided by the mean 0.87 μm reflectance of 4 × 4 250 m resolution pixels within a 1 km MODIS footprint (Liang et al., 2009). As in Liang et al. (2015), the data are further grouped into 2.5° latitude bins to capture latitudinal variations and 1° *SZA* bins within each latitude bin. The method of Liang et al. (2015) retrieves a *Fc* correction factor, such that *Re*_*corrected*_ *= Fc × Re*_*MODIS*_ *,* where *Re*_*corrected*_ is the “bias‐corrected” *Re* value and *Re*_*MODIS*_ is the original MODIS‐retrieved *Re* value. This is done for MODIS‐retrieved *Re* from the 1.6, 2.1, and 3.7 μm spectral channels, thus a separate *Fc* value is produced for each channel. For each latitude bin, *Fc* across all available *SZA* bins are retrieved, from which the mean, $\bar{\mathrm{Fc}}$, and standard deviation, *σ*_*Fc*_, are calculated for the upper and lower bounds of estimated *Fc*. The reported $\bar{\mathrm{Fc}}$ and *σ*_*Fc*_ therefore are both dependent on τ, *H*_*σ*_, latitude, month, and MODIS channel.

Along with stratifying the data by latitude, we also experimented with a bias‐correction approach using *SZA*, rather than latitude, as a dependent variable along with curve fitting rather than binning for the correction factors. The two approaches showed similar global distribution of bias‐corrected monthly‐mean *Re* values (section 3 below), with an overall global RMS difference of 0.6 μm and essentially unbiased. We present the latitude‐binning approach because (1) it is more tightly tied to the region, whereas the same *SZA* in a given month for Terra samples can occur both north and south of the solar equator, and (2) it provides *σ*_*Fc*_ used for error propagation. Further characterization by cloud regime is naturally captured by using *H*_*σ*_ and τ as dependent variables for the bias correction. We also examined the difference of stratifying by using both *H*_*σ*_ and τ against using only *H*_*σ*_ or τ. Our results (not shown) indicate that the inclusion of *H*_*σ*_ in the stratification process captures significantly more of the variability across the globe than stratification by τ alone.

Since some latitudes do not contain the required MISR‐observed scattering angles around the rainbow, $\bar{\mathrm{Fc}}$ is linearly interpolated between latitude bins with valid retrievals (Liang et al., 2015); these latitude bins are identified in the figures in section 3. For January, camera pairs were restricted to only adjacent neighboring MISR camera pairs (i.e., An‐A, A‐B, B‐C, and C‐D camera pairs for forward and aft directions). In July, far fewer latitude bins had adjacent MISR cameras observing scattering angles on both sides of the rainbow dip, so non‐adjacent camera pairs were also used for *Fc* retrievals at the expense of producing more widely separated upper and lower bound estimates of the MODIS *Re* bias (Liang et al., 2015).

Following the data stratification described above, we retrieve *Fc* for each τ‐*H*_*σ*_ bin to provide bias correction for each 2.5° latitude and longitude grid:

(1) Recorrectlatloni=Fc¯lati×ReMODISlatloni,

where *Re*_*correct(lat,lon)*_ is the corrected mean *Re* value and *Re*_*MODIS(lat,lon)*_ is the mean MODIS *Re* value for each 2.5° grid, ${\bar{\mathrm{Fc}}}_{\left(\mathrm{lat}\right)}$ is the upper or lower bound mean *Fc* for each 2.5° latitude bin, and *i* is the index for each τ‐*H*_*σ*_ bin (*i* = 1, 2, … , 35). For a given month, the final bias‐corrected monthly‐mean *Re* for each 2.5° grid point is weighted by the number of *Re* samples:

(2) Rew_correctlatlon=∑i=135Nlatloni∑i=135Nlatloni×Recorrectlatloni,

where *Re*_*w_correct(lat,lon)*_ is the weighted‐mean corrected *Re* for each 2.5° grid and *N*_*(lat,lon)*_ *(i)* is the number of *Re* samples that passed the quality control for each 2.5° grid for bin *i*.

The standard error in *Re*_*w_correct(lat,lon)*_ can be shown to equal:

(3) σSEcorrectlatlon=∑i=135Nlatloni∑i=135Nlatloni·σlatinlati·ReMODISlatloni2,

where $\sigma_{SE_{\text{correct}}\left(\mathrm{lat},\mathrm{lon}\right)}$ is the standard error of *Re*_*w_correct(lat,lon)*_, *σ*_(*lat*)_(*i*) is the standard deviation of upper or lower bound $\bar{\mathrm{Fc}}$ at 2.5° latitude resolution, and *n*_(*lat*)_ *(i)* is the number of *Fc* samples at 2.5° latitude resolution for bin *i*.

## 3 Results

### 3.1 Bias‐Corrected MODIS *Re*

**Figure 1:**
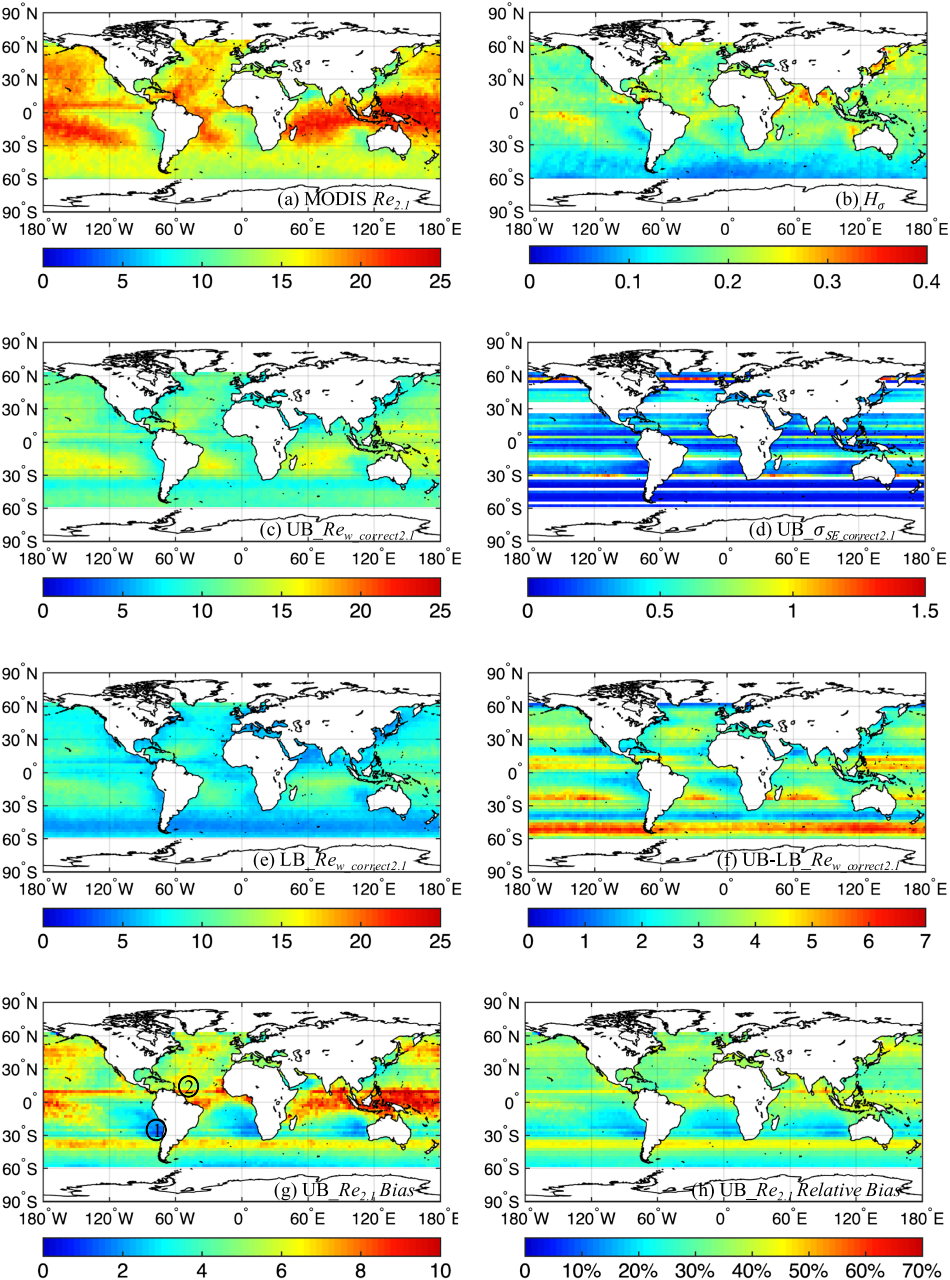
For January (2001–2008): (a) mean MODIS *Re*_*2.1*_ (μm) within MISR nadir camera swath; (b) mean *H*_*σ*_; (c) upper bound estimate of *Re*_*w_correct2.1*_ (μm); (d) standard error of the upper bound *Re*_*w_correct2.1*_ (μm); (e) lower bound estimate of *Re*_*w_correct2.1*_ (μm); (f) difference between the upper and the lower bound estimate of *Re*_*w_correct2.1*_ (μm); (g) upper bound estimates of mean MODIS *Re*_*2.1*_ bias (μm); and (h) upper bound estimates of mean MODIS *Re*_*2.1*_ relative bias (%). Latitude bands missing *Fc* retrievals are shown in white in (d), hence indicating that those regions are processed with interpolated *Fc* values in (c) and (e) as discussed in text.

**Figure 2:**
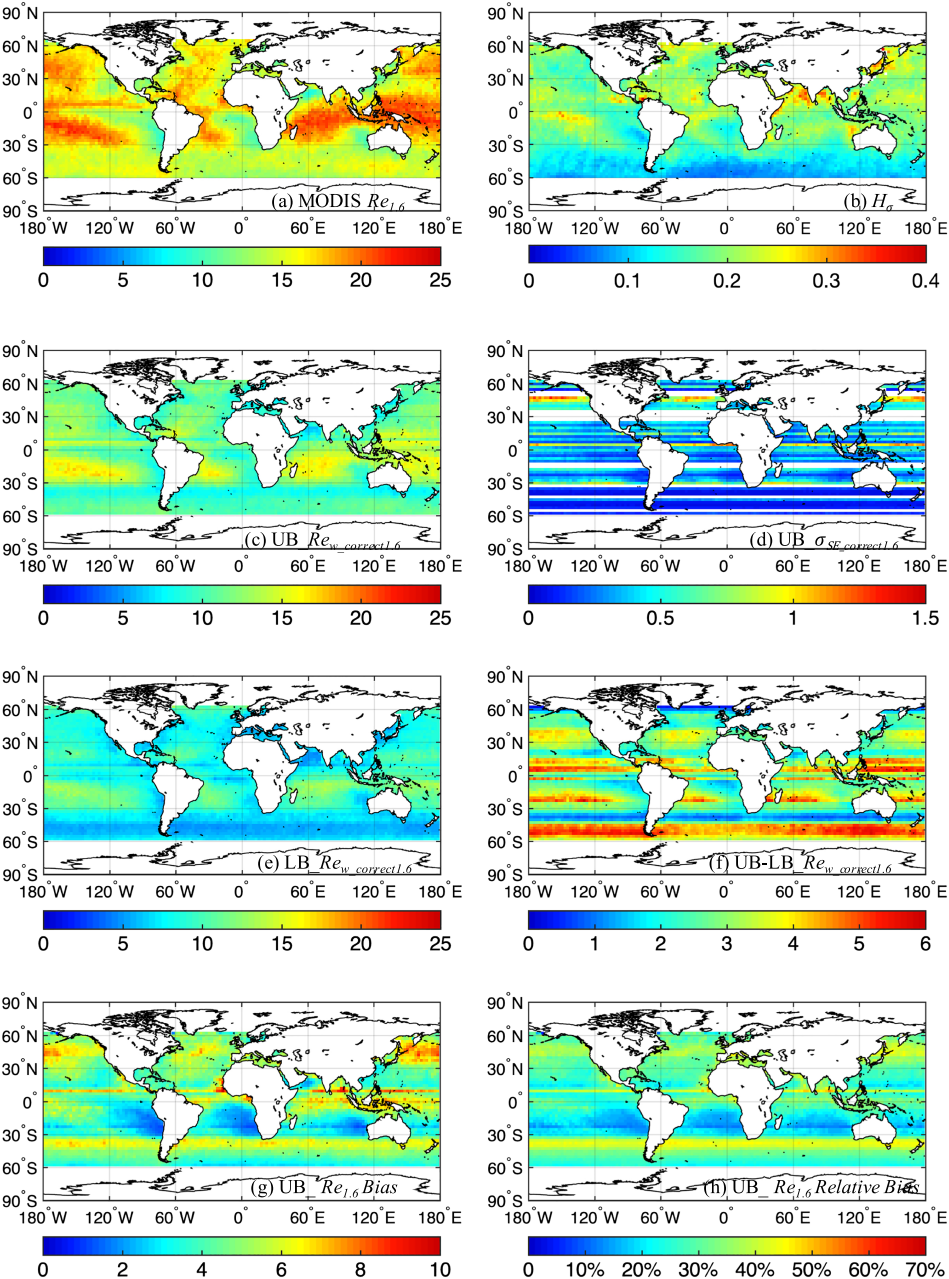
Similar to Figure 1 but for *Re*_*1.6*_.

**Figure 3:**
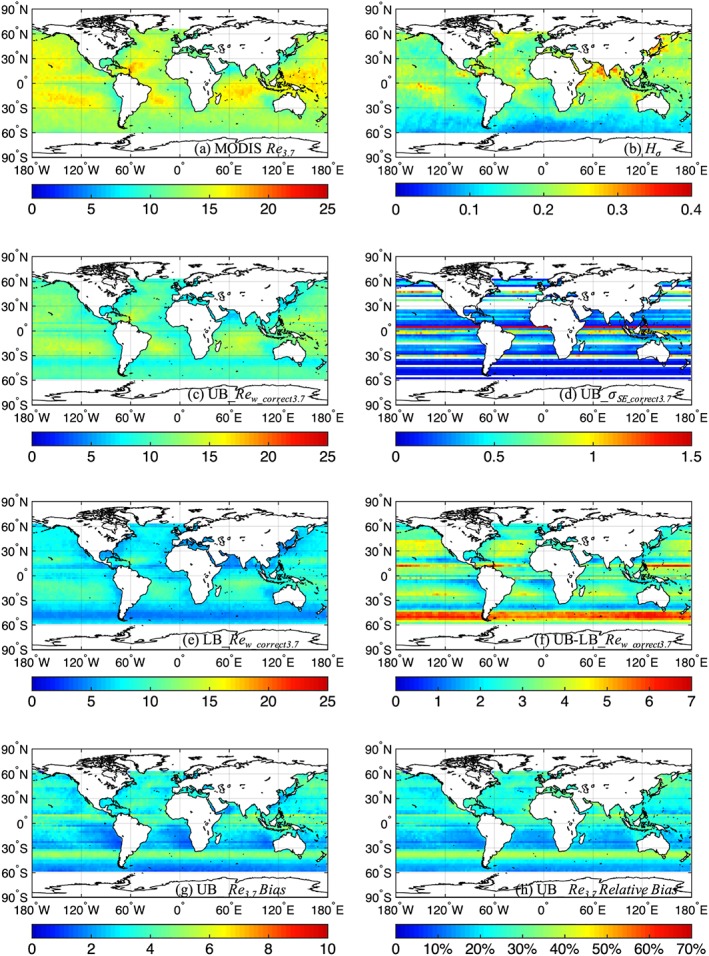
Similar to Figure 1 but for *Re*_*3.7*_.

Figure 1 shows our examination of *Re*_*2.1*_ for the month of January averaged between 2000 and 2008. Figure 1 includes the monthly mean of MODIS *Re*_*2.1*_, *Re*_*w_correct2.1*_ using the upper bound of *Fc* (minimum estimate of the bias) and lower bound of *Fc* (maximum estimate of the bias), the difference between upper and lower bound estimates, the minimum MODIS *Re*_*2.1*_‐bias based on the upper bound of *Fc*, and *H*_*σ*_—all sampled within the MISR nadir‐camera swath. Results for *Re*_*1.6*_ and *Re*_*3.7*_ are provided in Figures 2 and 3, respectively. Figures 1a, 1c, and 1e reveal large differences between the MODIS *Re*_*2.1*_ and *Re*_*w_correct2.1*_: The range of monthly mean *Re* values for MODIS *Re*_*2.1*_ is ~10 to 25 μm, while it is ~4 to 13 and ~5 to 17 μm for the lower and upper bounds of *Re*_*w_correct2.1*_, respectively. These produce area‐weighted, global (60°N to 60°S; ocean‐only) mean values of 16.6, 11.2, and 7.9 μm for MODIS *Re*_*2.1*_, upper bound *Re*_*w_correct2.1*_, and lower bound *Re*_*w_correct2.1*_, respectively. The standard error (Figures 1d) for the upper bound ranges from ~0.1 to 1.8 μm, with larger errors primarily due to regions with lower sampling in *Fc* retrievals (latitude bands missing *Fc* retrievals are shown in white, indicating that those regions are processed with interpolated *Fc* values as discussed in section 2). Similar values are had for the standard errors for the lower bound *Re*_*w_correct2.1*_ (not shown). These error estimates are mostly much smaller than the differences between the upper and lower bound estimates of *Re*_*w_correct2.1*_ shown in Figure 1f. The area‐weighted, global mean difference between upper and lower bound estimate of *Re*_*w_correct2.1*_ is 3.2 μm, with regional differences ranging from ~1 to 6 μm. In the following discussion, we mainly focus on the bias associated with the upper bound *Re*_*w_correct2.1*_ estimates (which gives minimum estimates of the bias in *Re*_*2.1*_ in Figure 1g)—the actual bias can in fact be larger, especially in regions where there are large differences between the upper and lower bound estimates of *Re*_*w_correct2.1*_. For example, around 35°S to 45°S, the bias in *Re*_*2.1*_ is tightly constrained by the upper and lower bounds to within ~1 to 3 μm (Figure 1f), with an upper bound estimate of *Re*_*w_correct2.1*_ ~7 to 9 μm. Around 45°S to 60°S, however, the bias in *Re*_*2.1*_ is less well constrained at ~4 to 6 μm (Figure 1f), with an upper bound estimate of *Re*_*w_correct2.1*_ ~10 to 12 μm. This zonal feature of the bias is further discussed below.

Figures 1g and 1h present the minimum bias (the difference between MODIS *Re*_*2.1*_ and the upper bound estimate of *Re*_*w_correct2.1*_) in terms of absolute mean values and relative values, respectively. Figure 1g reveals that the estimated bias in *Re*_*2.1*_ has a strong dependence on cloud regime, ranging from ~1 to 3 μm in regions with more homogeneous clouds (e.g., marine‐stratocumulus regions off the west coasts of South America, Africa, and Australia) to ~8 to 10 μm in regions with more heterogenous clouds (e.g., 5°S to 10°N in the western Pacific Ocean). This is consistent with our knowledge that greater 3‐D radiative effects that cause, on average, larger biases in the retrieval of *Re* using the bispectral technique occur for more heterogeneous clouds (e.g., Marshak et al., 2006). This is also reflected when compared to *H*_*σ*_ in Figure 1b, particularly in regions where differences in upper and lower bound estimates of *Re*_*w_correct2.1*_ are small (Figure 1f). In terms of relative bias (the ratio between the absolute bias and the MODIS *Re*_*2.1*_), Figure 1h shows that regions with large absolute biases do not necessarily correspond to regions with large relative biases. We see large bias (~40 to 60%) in regions around 30°S to 45°S, 5°S to 10°N, and 40°N to 50°N, and still small bias (~15 to 20%) in the coastal marine‐stratocumulus regions. Some zonal striping is evident in Figures 1c–1h. These may arise from several sources, including the smaller number of samples for some latitude bins, the zonal nature of solar zenith angles tied to Terra's 10:30 a.m. equator crossing time, and, to some degree, the zonal nature of the general circulation of the atmosphere.

Similar to Figure 1, Figures 2 and 3 show the correction for *Re*_*1.6*_ and *Re*_*3.7*_, respectively, for the month of January averaged between 2000 and 2008. Along with Figures 1a, Figures 2a and 3a reveal clear discrepancies between the original MODIS *Re*_*1.6*_, *Re*_*2.1*_, and *Re*_*3.7*_: the range of monthly mean *Re* values for MODIS *Re*_*1.6*_, *Re*_*2.1*_, and *Re*_*3.7*_ is ~9 to 22, ~10 to 25, and ~8 to 18 μm, respectively. After applying the bias correction procedures, much more consistent estimates among the three channels are revealed: with monthly mean *Re*_*w_correct*_ values generally within ~1 to 2 μm among the three channels. The area‐weighted, global mean values for MODIS *Re*, upper bound *Re*_*w_correct*_, and lower bound *Re*_*w_correct*_ for the three spectral channels are provided in Table 1. The difference between the upper and lower bound estimates of *Re*_*w_correct1.6*_ (Figure 2f) and *Re*_*w_correct3.7*_ (Figure 3f) are comparable to that of *Re*_*w_correct2.1*_ (Figure 1f) both in terms of spatial patterns and magnitudes. Figure 2 shows that the upper bound estimates of *Re*_*1.6*_ bias range from ~1 to 9 μm (Figure 2g), corresponding to ~15 to 60% in relative *Re*_*1.6*_ bias (Figure 2h). However, Figure 3 shows that the upper bound estimates of *Re*_*3.7*_ bias ranges from ~1 to 6 μm (Figure 3g), corresponding to ~12 to 45% in relative *Re*_*3.7*_ bias (Figure 3h), which is somewhat smaller than the upper bound estimates of *Re*_*2.1*_ bias and *Re*_*1.6*_ bias. This is consistent with the findings in Zhang and Platnick (2011) that *Re*_*3.7*_ is less susceptible to 3‐D radiative effects compared to the other two channel retrievals. Further discussion on spectral channel differences of retrieved and corrected MODIS *Re* are discussed in section 3.3.

**Table 1:** January Global Mean MODIS *Re*, Upper and Lower Bound Estimates of *Re*_*w_correct*_

MODIS channel	MODIS *Re* global mean	UB_*Re*_*w_correct*_ (standard error)	LB_*Re*_*w_correct*_ (standard error)
2.1 μm	16.6 μm	11.2 μm (0.3 μm)	7.9 μm (0.4 μm)
1.6 μm	16.1 μm	11.4 μm (0.4 μm)	8.4 μm (0.4 μm)
3.7 μm	13.8 μm	10.5 μm (0.4 μm)	7.4 μm (0.5 μm)

**Figure 4:**
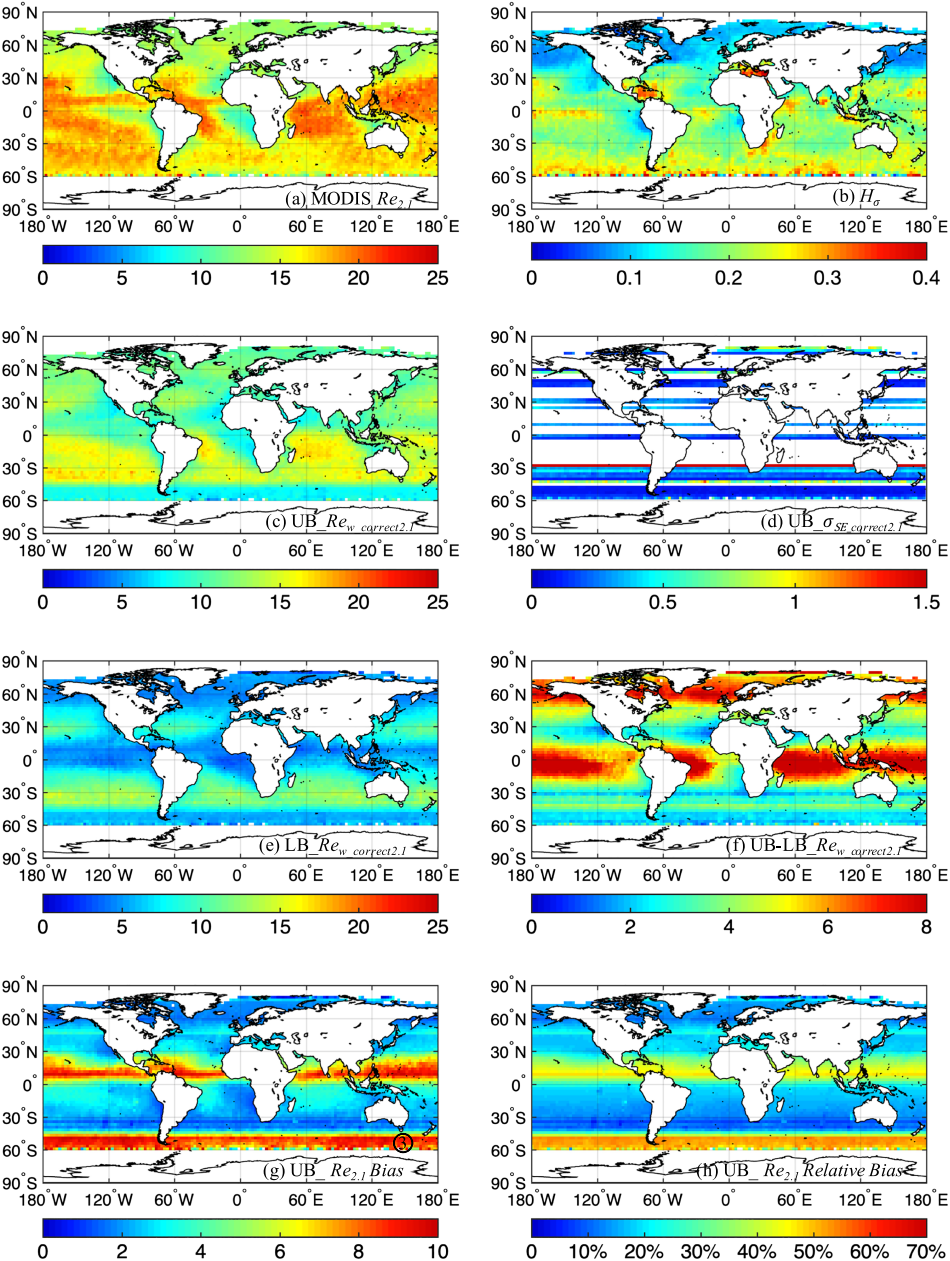
Similar to Figure 1 but for July (2001–2008).

Figure 4 displays the same information as Figure 1, but for July. Note that more latitudes have missing *Fc* retrievals, as indicated in Figures 4d, compared to January owing to less favorable scattering angles sampled by MISR for our technique (section 2). Figure 4a reveals the July monthly mean *Re* values for MODIS *Re*_*2.1*_ is ~9 to 21 μm, while it is ~3 to 14 and ~6 to 17 μm for the lower and upper bounds of *Re*_*w_correct2.1*_, respectively, after bias correction. The area‐weighted, global mean values for July are 16.3, 12.1, and 7.6 μm for MODIS *Re*_*2.1*_, upper bound *Re*_*w_correct2.1*_, and lower bound *Re*_*w_correct2.1*_, respectively. As noted above, the standard error for the upper bound estimates of *Re*_*w_correct2.1*_ in July have more latitudes with missing *Fc* retrievals, and the magnitude of the standard error is consistent with that of the January estimates. The difference between the upper and lower bound estimates of *Re*_*w_correct2.1*_ for July, however, reveals significant differences when compared to the January results: From Figure 4f, we observe large upper and lower bound differences of up to ~5 to 9 μm around 10°N to 15°S and 50 to 70°N, and much closer upper and lower bounds of ~1 to 4 μm around 15 to 30°N and 25 to 60°S. The large upper‐lower bound differences coincide with the latitudes that have missing *Fc* retrievals, which indicates that the lower sampling at the latitude boundaries of the missing *Fc* retrievals simply leads to larger differences between upper and lower bounds.

As in January, regional overestimates in the mean MODIS *Re*_*2.1*_ of at least ~1 to 10 μm (at the upper bound estimates of *Re*_*w_correct2.1*_) are observed for July (Figure 4g), but with different regional patterns owing to differences in the prevailing cloud heterogeneities (Figures 1b vs. 4b) and solar zenith angles. Specifically, the relative biases in Figures 1h and 4h reveal interesting patterns: Large relative bias along ~10°N is consistent with convective clouds along the ITCZ, and the large biases in the tropics spreading southward in January and northward in July are in line with the seasonal shift in the strength of the Hadley cell (e.g., Dima & Wallace, 2003). This also correlates well with tropical *H*_*σ*_ values in Figures 1b and 4b, since larger *Re* biases are expected to occur for more heterogeneous clouds. At middle to high latitudes in the Southern Hemisphere, January shows a band of large *Re* biases between ~35°S and 40°S (large *H*_*σ*_ in Figure 1b) and small *Re* biases within ~45°S to 60°S (small *H*_*σ*_ in Figure 1b), indicating that in moderately high *SZA*s, the texture of clouds (quantified by *H*_*σ*_) dominates the zonal variations in *Re* bias more than *SZA* effects. In July, however, low *Re* biases are observed from ~30°S to 45°S, while bias increase substantially between 45°S and 60°S despite no corresponding increase in the moderately large *H*_*σ*_ values, indicating the dominant effects of large *SZA*s (e.g., from shadows) on the gradient in the bias. Such rapid increases in retrieval errors with increasing *SZA*s larger than ~70° for heterogeneous clouds have been discussed in other studies (e.g., Grosvenor & Wood, 2014; Loeb et al., 1997). One curious feature in Figure 4h is that the *Re*_*2.1*_ bias for July is low in ~30°S to 45°S (*SZA* ~55° to 70°) relative to regions to the north and south. This may be due to competing 3‐D‐radiative pathways that produce a minimum in retrieval errors at midrange *SZA*s (e.g., Kato & Marshak, 2009; Várnai & Davies, 1999). Hints of this also appear in the Northern Hemisphere ~20°N to 35°N for January (*SZA* ~50° to 64°) but is made difficult to see due to large variations in *H*_*σ*_. Overall, the Northern Hemisphere in January shows *Re*_*2.1*_ bias variations that are consistent with variations in large *SZA*s and *H*_*σ*_, while in July the *Re*_*2.1*_ bias variations are dominated by textural variations rather than variations in moderately small *SZA*s. Similarly, over the Southern Hemisphere in July, the *Re*_*2.1*_ bias variations under low Sun are associated with variations in large *SZA*s and *H*_*σ*_, whereas in January the *Re*_*2.1*_ bias variations in moderately small *SZA*s are mostly dominated by textural variations.

### 3.2 Comparison to Other Data Sets

The *Re* biases reported in Figures 1g and 4g compare very well with the few existing in situ validation studies of MODIS *Re*_*2.1*_. For example, using data from the VAMOS Ocean‐Cloud‐Atmosphere‐Land Study Regional Experiment (VOCALS‐REx) in the Southeast Pacific during October to November 2008 (region ① in Figure 1g), Painemal and Zuidema (2011) showed an ~1 to 2 μm bias in MODIS *Re*_*2.1*_; from the Rain in Cumulus over the Ocean (RICO) field campaign from November 2004 to January 2005 (region ② in Figure 1g), Haney (2013) showed MODIS *Re*_*2.1*_ biases ranging from ~7 to 12μm, and aircraft observations in the southern ocean (region ③ in Figure 4g) from June to October in 2013 to 2015, Ahn et al. (2018) showed an overestimate in MODIS *Re*_*2.1*_ of ~13 μm on average for nondrizzling clouds. All of these studies line up nicely with the regional *Re*_*2.1*_ biases shown in Figures 1g and 4g. We also note that Miles et al. (2000) provided a review of field observations of marine liquid water clouds over the past 50 years indicating *Re* typically ranging from ~4 to 15 μm, which is in line with *Re*_*w_correct2.1*_. Similar conclusions are drawn in comparison with *Re*_*w_correct1.6*_ and *Re*_*w_correct3.7*_.

**Figure 5:**
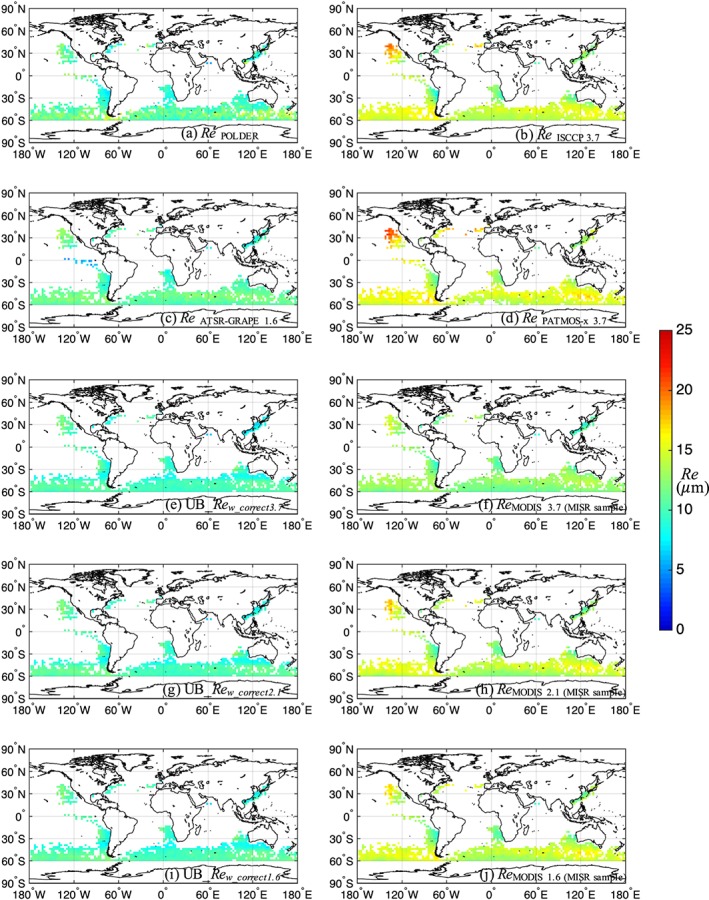
(a) POLDER *Re*, (b) ISCCP *Re*_3.7_, (c) ATSR‐GRAPE *Re*_1.6_, (d) PATMOS‐x *Re*_3.7_, (e) upper bound estimates of *Re*_*w_correct3.7*_, (f) MODIS *Re*_*3.7*_, (g) upper bound estimates of *Re*_*w_correct2.1*_, (h) MODIS *Re*_*2.1*_, (i) upper bound estimates of *Re*_*w_correct1.6*_, and (j) MODIS *Re*_*1.6*_ for January.

We also compare the upper bound estimates of *Re*_*w_correct*_ reported in Figures 1, 2, and 3 against available *Re* products derived from various satellites (Figure 5), specifically the Pathfinder Atmospheres‐Extended (PATMOS‐x; Heidinger et al., 2014) from 2002–2008, the International Satellite Cloud Climatology Project (ISCCP; Rossow & Schiffer, 1991) from 1992–1999, the Along‐Track Scanning Radiometers‐Global Retrieval of ATSR Cloud Parameters and Evaluation (ATSR‐GRAPE; Sayer et al., 2011) from January 2003–2009, and the Polarization and Directionality of the Earth's Reflectances (POLDER; Deschamps et al., 1994) from January 2006–2013. All data but POLDER were retrieved from the GEWEX Cloud Assessment Database (Stubenrauch et al., 2013). For POLDER, only Level 2 *Re* retrievals with a quality index >2.2 (Bréon & Doutriaux‐Boucher, 2005) are averaged to a 2.5° resolution grid to ensure fair global coverage and sufficient sampling for analysis. Stubenrauch et al. (2012) has shown large differences in *Re*_*2.1*_ among all these datasets with regional differences of around −7 to +9 μm in monthly means, but without exploring why they are different. Here, we focus on regions with POLDER sampling only since it is believed that POLDER provides superior retrievals owing to its advanced polarimetric approach, which is less sensitive to biases arising from 3‐D effects (Bréon & Doutriaux‐Boucher, 2005). All satellite retrievals are sampled to a 2.5° resolution grid and filtered to only include the grids that have sufficient POLDER coverage.

Figure 5 reveals that, despite the independent nature of the various satellite instruments and the differences in sampling, a clear pattern emerges when organized by retrieval methods (bispectral methods on the right and other methods on the left): Panels in the left column share very similar regional patterns of *Re*, while panels on the right also show similar regional patterns but with much larger regional differences among each other. When compared with POLDER *Re*, the mean difference of monthly averages and RMS differences (in bracket) are provided in Table 2. The ~3 μm difference of *Re*_MODIS2.1_ compared to POLDER *Re* is close to the ~2 μm difference reported in Bréon and Doutriaux‐Boucher (2005), where the ~1 μm discrepancy may be due to different data collections (MODIS Collection 4 vs. 6), our sampling to within the MISR swath, and differences in quality control procedures. After applying our bias correction procedures, the upper bound estimates of *Re*_*w_correct2.1*_ is now much more consistent with POLDER *Re* (mean difference of approximately −0.77 μm), even though in some regions (particularly in the Southern Ocean) POLDER *Re* have slightly larger values than *Re*_*w_correct2.1*_; further limiting the POLDER *Re* to only include grids with standard error of the mean <1.5 μm greatly reduces such differences. It is encouraging that the *Re* maps in the left column of Figure 5 come from independent retrieval techniques (i.e., polarization, multi‐angle, and bias correction), and with different spectral channels (i.e., 1.6, 2.1, and 3.7 μm), yet produce consistent results. Spectral channel differences are discussed in more detail in section 3.3.

**Table 2:** Global Mean Difference of January Monthly Averages and RMS Difference Compared to POLDER *Re*

*Re* product	Mean difference	RMS	*Re* product	Mean difference	RMS
*Re*_ATSR‐GRAPE_	+0.06 μm	+1.73 μm	*Re*_ISCCP_	+3.64μm	+4.02μm
*UB_Re*_*w_correct3.7*_	−1.00 μm	+1.87 μm	*Re*_MODIS3.7_	+4.34μm	+4.73μm
*UB_Re*_*w_correct2.1*_	−0.77 μm	+1.72 μm	*Re*_MODIS2.1_	+1.6μm	+2.33μm
*UB_Re*_*w_correct1.6*_	−0.81 μm	+1.75 μm	*Re*_MODIS1.6_	+3.31μm	+3.72μm
			*Re*_PATMOS‐x_	+3.32μm	+3.65μm

As a final means of comparison, we turn to global models. Ban‐Weiss et al. (2014) showed that MODIS *Re*_*2.1*_ over oceanic regions are globally ~6 μm higher than simulated *Re* from several General Circulation Models (GCMs). While large differences exist in regional patterns of *Re* among the GCMs, none match the regional patterns of MODIS *Re*. In comparison to our estimates of *Re*_*w_correct2.1*_, however, simulated *Re* from the GCMs shown in Ban‐Weiss et al. (2014) generally falls within the lower bound *Re*_*w_correct*_ (~4 to 13 μm) and the upper bound *Re*_*w_correct*_ (~5 to 17 μm). Furthermore, we note that the *Re* simulated by the Geophysical Fluid Dynamics Laboratory‐Atmosphere Model 3 (AM3) GCM shown in Ban‐Weiss et al. (2014) exhibit *Re* patterns and values similar to *Re*_*w_correct2.1*_.

### 3.3 *Re*_*w_correct*_ Channel Differences

Platnick (2000) showed that the retrieved *Re* by the bispectral method is a convolution of the vertically varying *Re* and a radiative weighting function that is spectrally dependent. The retrieved *Re*_*3.7*_ is more strongly weighted toward *Re* values that are nearer to cloud top as compared to *Re*_*2.1*_ or *Re*_*1.6*_ because of stronger absorption by liquid water at 3.7 μm. Using a wide range of idealized vertical profiles of *Re*, Platnick (2000) showed that the influence of vertical variations in cloud microphysics may result in *Re* differences of up to 1.5 μm among these three spectral channels. This has led to the possibility of retrieving the vertical profile of *Re* using multiple channels (e.g., Chang & Li, 2002, 2003; Chen et al., 2008). However, 3‐D effects also impact the channel retrievals to various degrees. For example, Zhang et al. (2012) concluded that cloud horizontal heterogeneity can lead to substantial bias between *Re*_*2.1*_ and *Re*_*3.7*_ (up to ~10 μm for highly heterogeneous cumulus clouds), making the interpretation of vertical variability of *Re* difficult to ascertain.

**Figure 6:**
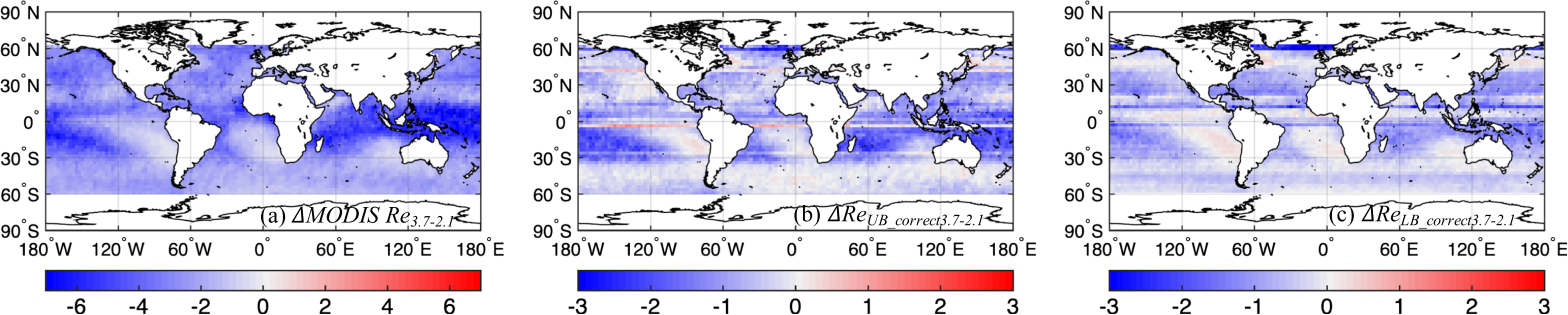
(a) Difference of MODIS *Re*_*3.7*_ and *Re*_*2.1*_ for January 2001–2008 (*Δ* *R*e_*3.7‐2.1*_; μm). (b) Difference of the upper bound *Re*_*w_correct3.7*_ and *Re*_*w_correct2.1*_ (*Δ* *Re*_*UB_correct3.7‐2.1*_) for January 2001–2008 (μm). (c) Difference of the lower bound *Re*_*w_correct3.7*_ and *Re*_*w_correct2.1*_ (*Δ* *Re*_*LB_correct3.7‐2.1*_) for January 2001–2008 (μm).

Figure 6 shows the difference between the mean MODIS *Re*_*2.1*_ and MODIS *Re*_*3.7*_ sampled within the MISR nadir‐camera swath (hereafter *Δ* *Re*_*3.7‐2.1*_), the difference between the upper bound estimates of *Re*_*w_correct2.1*_ and *Re*_*w_correct3.7*_ (hereafter *Δ* *Re*_*UB_correct3.7‐2.1*_), and the lower bound estimates of *Re*_*w_correct2.1*_ and *Re*_*w_correct3.7*_ (hereafter *Δ* *Re*_*LB_correct3.7‐2.1*_) for January. Note the difference in scales in Figures 6. Similar to the findings in Zhang and Platnick (2011), Figure 6a shows substantial *Δ* *Re*_*3.7‐2.1*_ differences between the two channel retrievals, ranging from approximately –6 μm in the more cumuliform cloud regions to approximately –1 μm in more stratiform cloudy regions. This range drops substantially for *Δ* *Re*_*UB_correct3.7‐2.1*_: approximately –2 μm in the cumuliform cloudy regions to slightly positive (up to +0.6 μm) in the stratiform cloudy regions, demonstrating that the bias‐correction procedure effectively reduces the disparity between different MODIS spectral *Re* retrievals to values that are more in line with the simulated values given in Platnick (2000). *Δ* *Re*_*LB_correct3.7‐2.1*_ (Figure 6c) shares the same pattern as *Δ* *Re*_*UB_correct3.7‐2.1*_, with slightly lower magnitude of approximately −1.2 μm in the cumuliform cloudy regions. The standard error (not shown) associated with *Δ* *Re*_*UB_correct3.7‐2.1*_ and *Δ* *Re*_*LB_correct3.7‐2.1*_ are generally within ~0.2 μm in the homogeneous cloud regions and are within ~0.6 μm in the heterogeneous cloud regions, with a few latitudes reaching ~1.5 μm due to low sampling.

It is evident from Figure 6 that even after the bias correction procedure, the spatial patterns of *Δ* *Re*_*3.7‐2.1*_ are well preserved in *Δ* *Re*_*UB_correct3.7‐2.1*_ and *Δ* *Re*_*LB_correct3.7‐2.1*_ and that they are dominated by the spatial variability of cloud type (e.g., Stubenrauch et al., 2012). The dependence of *Δ* *Re*_*3.7‐2.1*_, *Δ* *Re*_*UB_correct3.7‐2.1*_, and *Δ* *Re*_*LB_correct3.7‐2.1*_ on cloud regime strongly indicates large differences in the vertical variation of *Re* between cumuliform and stratiform cloud regimes near cloud tops. Since negative (positive) values in *Δ* *Re*_*UB_correct3.7‐2.1*_ and *Δ* *Re*_*LB_correct3.7‐2.1*_ indicate decreasing (increasing) *Re* with increasing altitude near cloud top, our results indicate that cumuliform clouds show much stronger decreases in *Re* with increasing altitude near cloud tops as compared to stratiform clouds, assuming that such vertical variations are monotonic over much of the depth over which the weighting is significant (e.g., the first few optical depths as measured from cloud top). This may perhaps be due to the nature of mixing within these clouds, being dependent on the nature of convection (e.g., driven by cloud top radiative cooling for stratocumulus vs. warming from below for cumulus), the horizontal and vertical cloud scales, and other meteorological factors that govern the life cycle of clouds. Further investigation is warranted.

## 4 Conclusion

We employed the method of Liang et al. (2015) to estimate upper and lower bound estimates in the bias of MODIS *Re* of marine liquid clouds but stratified by *SZA*, *H*_*σ*_, and *τ*, to produce bias‐corrected *Re* at regional scales. Using 8 years of January and July MISR and MODIS fusion data, we reported lower and upper bound estimates of bias‐corrected mean MODIS *Re*_*2.1*_ ranging from ~4 to 13 μm and ~5 to 17 μm, respectively, depending on the cloud regime and *SZA*. Our results compared favorably to existing in situ validation for MODIS *Re* (e.g., Ahn et al., 2018; Haney, 2013; Painemal & Zuidema, 2011), to surveys of marine *Re* from aircraft (Miles et al., 2000), to satellite retrievals from POLDER and ATSR in regions sampled by POLDER, and to GCM generated *Re* reported in Ban‐Weiss et al. (2014), in particular, those from the AM3 GCM. The spatial patterns observed in the *Re* biases are easily understood in terms of our understanding of global cloud type distributions and the impact of 3‐D radiative transfer through heterogeneous clouds on 1‐D retrievals of cloud properties (as discussed in section 3.1). The spatial patterns also match nicely with the measured deviations of the angular anisotropy of the radiation field sampled from MISR from 1‐D radiative transfer solutions reported in Di Girolamo et al. (2010).

Differences in bias‐corrected MODIS *Re* between *Re*_*3.7*_ and *Re*_*2.1*_ remain, ranging between −2 and +0.6 μm, compared to the original MODIS differences of −6 to −1 μm in regional monthly means. The spectral channel differences in bias‐corrected *Re* are more in line with the differences in spectral‐channel *Re* simulated by Platnick (2000) arising from vertical variations in *Re*. Difference between the bias‐corrected MODIS *Re*_*3.7*_ and *Re*_*2.1*_ showed a clear dependence on cloud regimes, suggesting very different vertical variations of *Re* near cloud top between cloud regimes.

The bias‐corrected MODIS *Re* presented herein should give way to better estimates of droplet number concentration (*N*_d_; Grosvenor et al., 2018) and liquid water path (LWP; Zhou et al., 2016; Greenwald et al., 2018), since *N*_d_ and LWP derived from MODIS are actually derived from the MODIS‐retrieved *Re* and τ. Since we have not assessed the bias in τ, we cannot estimate the biases in *N*_*d*_ and LWP at this point. Still the fractional contribution of the *Re* error to LWP and *N*_*d*_ is significant, given that LWP ~ *Re* and *N*_*d*_ ~ *Re* ^‐5/2^ (e.g., Grosvenor et al., 2018). We anticipate that the bias‐corrected *Re* results may allow for a refined examination of studies on aerosol‐cloud interaction (e.g., Costantino & Bréon, 2010; Myhre et al., 2007).

As noted in section 2, we only considered the MODIS *Re* retrievals of liquid clouds that are within the MISR swath and passing the quality control procedures for MISR and MODIS data fusion. A total of 48.2% and 51.4% of all fully cloudy, valid MODIS retrievals are used in this study for the months of January and July, respectively. While our results are valid for those samples, we have not assessed their climatological representativeness (also true for the MODIS retrievals as discussed in Cho et al., 2015). Still, the climatology constructed from our samples does compare favorably to ATSR and POLDER (Figure 5), perhaps indicating that sample representativeness may not have a significant impact on the climatology presented herein.
